# Exploring human milk oligosaccharides: mechanisms linking gut function to cognitive development in human and pig physiology

**DOI:** 10.3389/fnut.2025.1700954

**Published:** 2025-12-12

**Authors:** Wentian Li, Allan Gunn, Xiaoming Zheng, Jingyi Ma, Nan Sheng, Bing Wang

**Affiliations:** 1 Gulbali Institute for Agricultural Water and Environment, Wagga Wagga, NSW, Australia; 2 School of Agricultural, Environmental and Veterinary Sciences, Faculty of Science and Health, Charles Sturt University, Wagga Wagga, NSW, Australia; 3 Nutrition Research Institute, Junlebao Dairy Group Co., Ltd., Shijiazhuang, China

**Keywords:** human oligosaccharides, gut microbiome, cognitive function, pig physiology, behavioral outcomes

## Abstract

Human milk oligosaccharides (HMOs) are emerging as key modulators of host physiology, with growing evidence supporting their role in shaping gut microbial communities and influencing neurocognitive outcomes. This review critically examines the impact of HMOs on gut health and behavioral responses, focusing on the complex relationship between HMOs and host physiology in both human and pigs. Given their anatomical, physiological, and microbiome similarities to humans, pigs serve as a valuable translational model for investigating the functional roles of HMOs. We summarize experimental methodologies employed in HMO research and highlight findings that demonstrate HMO-induced alterations in microbial diversity, gut integrity, and cognitive performance. Potential mechanisms of action, including gut–brain axis signaling, immune modulation, and microbial metabolite production, are explored. This review concludes by identifying current knowledge gaps and proposing future research directions aimed at elucidating HMO structure–function relationships, with implications for advancing both human nutrition and animal health.

## Introduction

Human Milk Oligosaccharides (HMOs) have emerged as central players in the complex interplay between nutrition and physiology(1). While their primary relevance lies in human infant nutrition, pigs serve as an excellent model for studying HMOs' functions in humans due to their close similarities in digestive systems, nutritional demands, and gut microbiomes (2). Their physiological responses and developmental processes closely align with those of humans, offering practical advantages for meaningful and significant translational implications in understanding the impact of HMOs on human health (3). Recognizing the nutritional value of HMOs, especially in breast milk, reveals a host of bioactive compounds that can affect gut health (4–6). The nutritional stories of HMOs unfold in the day of breastfeeding mothers and babies, where these oligosaccharides play a multifaceted role. Currently, more than two hundred different structures of HMOs have been identified, and every lactating woman can synthesizes a unique set of milk oligosaccharides (7).

In recent years, HMOs such as 2′-fucosyllactose (2′-FL) and lacto-N-neotetraose (LNnT) have received GRAS (Generally Recognized as Safe) status and been used in infant formula, the regulatory pathways for other HMOs are still underdeveloped. Although microbial fermentation and enzymatic synthesis of HMOs have shown great promise, these biotechnological approaches often suffer from low yields and require costly substrates and tightly controlled conditions (8, 9). Moreover, the purification and quality control of individual HMO species remain technically challenging, particularly when dealing with structurally similar isomers. Thus the application of HMOs has been significantly limited by high reagent costs, low production yields and structural complexity (10). The industry now is actively working to improve the efficiency of HMO production to reduce costs and ensure their inclusion in infant formula in an affordable and widely accessible manner. Although cow's milk contains bovine milk oligosaccharides (BMOs) that are structurally related to HMOs, they are far less abundant and structurally simpler. For this reason, researchers are incorporating HMOs into cow's milk–based formulas to better approximate the composition and biological functions of human breast milk (11, 12). While it remains unclear whether different HMOs exert their effects individually or in combination within the human body, this review emphasizes the function of HMOs and explores their potential to enhance gut health and cognitive function in both human and porcine physiology. This review examines the latest understanding of how specific HMOs impact cognitive function, gut health and behavior outcomes in both humans and pigs.

## Biological functions of HMOs

HMOs, unique to human breast milk, play a critical role in infant development. They are broadly classified into three major structural groups: neutral non-fucosylated, neutral fucosylated, and acidic (sialylated) HMOs (13). Numerous studies have shown that HMOs levels vary among women and during different stages of lactation (14). This variation is due to genetic factors, dietary influences, and other environmental conditions (7). The most abundant HMOs in most mothers' breast milk is 2′-FL, a trisaccharide composed of glucose, galactose and fucose. Its presence is found in mothers who are secretors—a trait determined by the activity of the FUT2 gene, which governs secretor status and is indirectly associated with the mother's ABO blood group (7, 15). Two large international studies have shown that 2′-FL is present in the milk of approximately 65–98% of lactating women across 21 countries, with concentrations ranging from 0.06 to 4.65 g/L (14, 16). This variability reflects differences in maternal secretor status, governed by the FUT2 gene, which determines the ability to synthesize α1,2-fucosylated HMOs such as 2′-FL. Human milk from non-secretor mothers, who lack active FUT2 gene expression, contains neutral HMOs such as Lacto-N-tetraose (LNT) and LNnT, but typically lacks fucosylated HMOs like 2′-FL (17, 18). However, all human milk contains sialylated HMOs, which include sialic acid residues—often considered important markers of immune and neurodevelopmental support (14, 19). HMOs are recognized as complex bioactive compounds essential for neonatal nutrition, forming a critical component of the nutritional foundation for newborns.

HMOs are important components of human milk that have shown various health benefits across different populations. 2′-FL is the first HMO to be included in commercial infant formulas. Clinical trials over the past two decades have consistently demonstrated that HMO supplementation, including 2′-FL, is safe and well tolerated in infants, children, and adults, with no serious adverse effects (20, 21). In infants, HMO supplementation has been shown to shape the gut microbiota, enhance immune function and protect against pathogens. It promotes gut microbiome composition, stool characteristics, and immune responses similar to those of breastfed infants, potentially reducing the incidence of infections and the risk of necrotizing enterocolitis (NEC) (22–26). HMOs primarily act as prebiotics, promoting the growth of beneficial bacteria such as *Bifidobacteria* as demonstrated in clinical trials (26). This, in turn, enhances gut health, supports immune function, and fosters a balanced gut microbiome. Additionally, HMOs serve as decoy structures, mimicking receptors on gut cells that pathogens (like harmful bacteria and viruses) typically bind to (27, 28). By doing this, HMOs prevent these harmful microorganisms from attaching to the gut lining, which helps protect against infections and supports overall gut health (25, 29). HMOs also act as soluble decoy receptors that inhibit pathogen adhesion to the intestinal epithelium; for instance, 2′-FL reduces the attachment of enteropathogenic E. coli to host cells (30). Despite these promising findings, the clinical relevance in humans of different ages and certain medical conditions remains unclear, necessitating further research to substantiate the health benefits and understand the long-term effects of HMO supplementation (31).

The pig has been an ideal animal model for learning the HMOs' function (1, 32, 33). Researchers have found that pigs fed HMOs show improved performance in object recognition tests and exhibit better long-term memory. Additionally, HMOs can prevent pathogen adhesion and may also play a role in enhancing long-term cognitive function (1). Although the precise mechanisms remain unclear, several hypotheses have been proposed to explain how HMOs may affect gut function and cognitive development as summarized below.

## Possible mechanisms by which HMOs influence gut function and cognitive development

**Figure 1:**
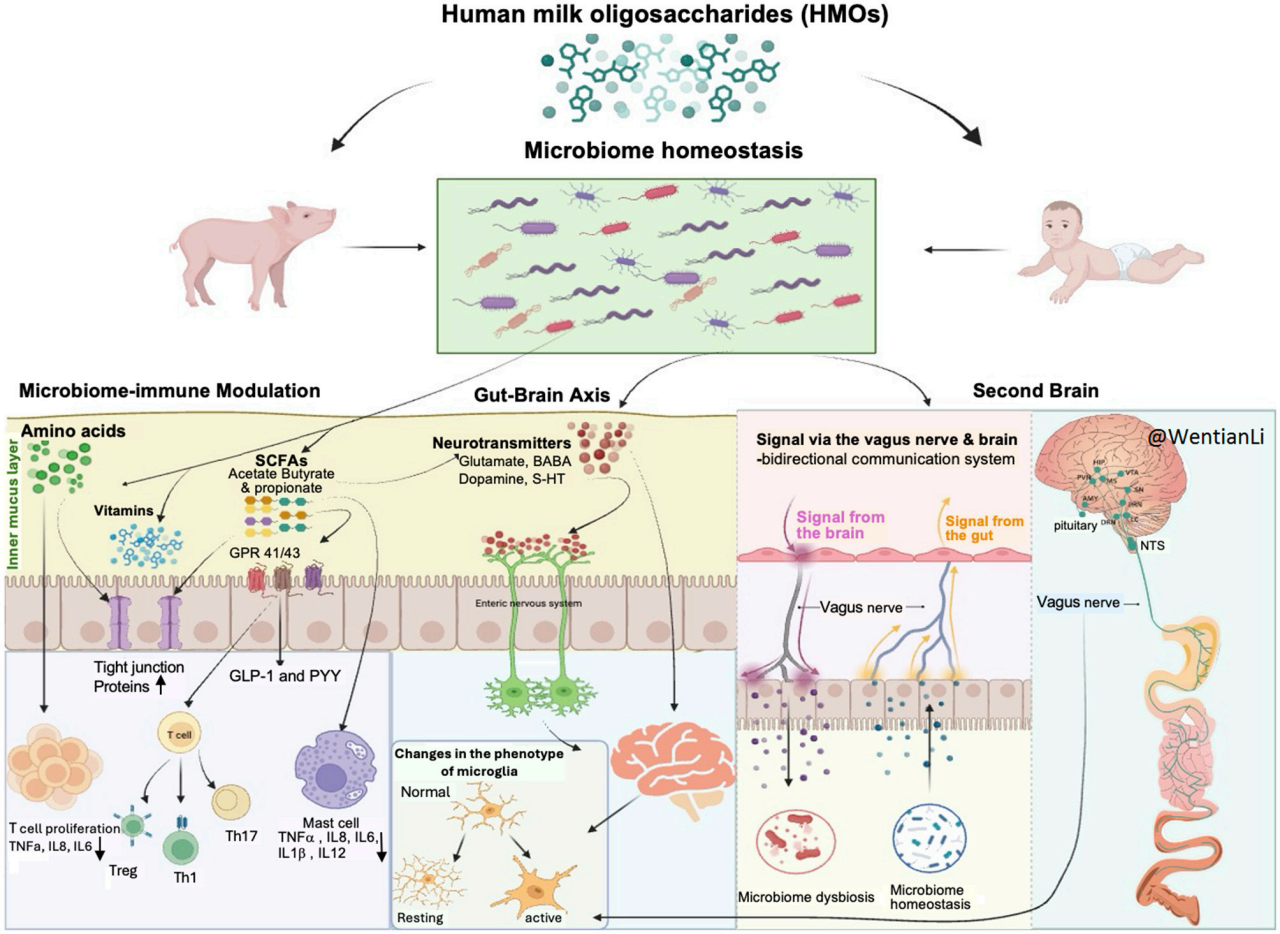
Potential mechanisms and interactions between gut microbiota and brain. (1) Microbiome-Immune Modulation: HMOs shape gut microbiota, modulating immune responses and producing metabolites (e.g., SCFAs, amino acids) that affect brain development. (2) Gut-Brain Axis: HMOs impact neural, immune, and hormonal pathways, influencing neurotransmitters like GABA and serotonin linked to cognition and emotion. (3) Second Brain Hypothesis: HMOs may act via the vagus nerve, enabling bidirectional gut-brain communication and supporting mental health. This figure was created using BioRender (https://biorender.com/), an online tool for creating scientific diagrams.

Currently, the connection between gut function and cognitive development is supported by several hypotheses. Figure 1 illustrates three major mechanisms through which HMOs may influence gut function and cognitive brain development: (1) microbiome–immune modulation, (2) the hypothalamic–pituitary–adrenal (HPA) axis, and (3) the vagus nerve/second brain pathway within the broader gut–brain axis.

*The Microbiome-Immune Modulation Hypothesis* proposes that HMOs may affect the gut microbiota, which in turn modulate the host's immune system, affecting brain development and cognitive function. An imbalance in gut microbes may lead to inflammation, which can subsequently impair cognitive function (34, 35). Moreover, gut-derived metabolites like short-chain fatty acids (SCFAs), B vitamins and amino acids can cross the blood-brain barrier (BBB) and directly influence brain function (36, 37). Certain amino acids, such as tryptophan, glutamine, and arginine, produced by the gut microbiota, alter T-cell activity and modulate the release of inflammatory cytokines, such as TNF-α, IL-8, and IL-6 (38). They also maintain gut barrier integrity by regulating tight junction proteins, which prevent toxins and pathogens from entering the bloodstream and inducing immunological reactions. For example, microbial fermentation of HMOs in the gut produces SCFAs, which offer various beneficial local and systemic effects, including anti-inflammatory properties and support for intestinal barrier function (27, 39). SCFAs such as acetate, propionate, and butyrate enhance immunological homeostasis by promoting T-cell differentiation (e.g., Treg, Th1, and Th17) and by stimulating the secretion of gut hormones GLP-1 (glucagon-like peptide-1) and PYY (peptide YY) through activation of G-protein-coupled receptors GPR41 and GPR43. The release of GLP-1 and PYY strengthens intestinal barrier integrity, reduces gut permeability, and indirectly modulates inflammatory signaling by lowering systemic endotoxin exposure. In addition, GLP-1 exerts anti-inflammatory effects on immune cells through inhibition of NF-κB signaling and cytokine production, while PYY helps maintain mucosal immune balance by influencing macrophage and dendritic cell activity. Together, these pathways link microbial SCFA metabolism to both metabolic and immune equilibrium within the gut–immune axis (40, 41). Furthermore, SCFAs modulate mast cell activity by engaging G-protein–coupled receptors such as GPR41, GPR43, and GPR109A, and by inhibiting histone deacetylases (HDACs), which together suppress mast cell activation and degranulation (42, 43). This results in reduced release of pro-inflammatory mediators, including TNF-α, IL-6, and IL-8, as well as histamine and proteases like tryptase (44). By limiting these mediators, SCFAs help maintain intestinal and systemic immune homeostasis, preventing excessive vascular permeability, leukocyte recruitment, and cytokine-driven inflammation. The downstream effect is reduced activation of microglia and protection against neuroinflammation, blood–brain barrier disruption, and excitotoxicity, thereby supporting immune–neural balance and cognitive function (45).

*The Gut-Brain Axis Hypothesis* is increasingly recognized and accepted. Within this framework, two major routes of communication have been proposed: (1) immune and metabolic modulation, and (2) endocrine regulation through the hypothalamic–pituitary–adrenal (HPA) axis. This hypothesis suggests that the involvement of HMOs in modulating the gut communicates with the brain through multiple pathways, including immune, neural and hormonal pathways.

Specific gut microbiomes influence brain function by regulating neurotransmitters such as gamma-aminobutyric acid (GABA), dopamine and serotonin, affecting memory, cognition, and emotional regulation (46). Beyond their direct effects on brain function, these neurotransmitters also influence the enteric nervous system (ENS) and regulate the activity of microglia in the central nervous system (CNS). Under certain circumstances, microglia—the brain's resident immune cells—can become activated, undergoing morphological alterations in response to neurotransmitter shifts regulated by the gut microbiota (47). In addition, gut microbes can modulate the HPA axis by influencing the release of corticotropin-releasing hormone (CRH), adrenocorticotropic hormone (ACTH), and cortisol, linking microbial activity to stress response, emotional regulation, and cognitive outcomes. This activation impacts neuroinflammatory pathways and neural plasticity, affecting brain health. Therefore, the microbiota-gut-brain axis appears bidirectionally, with signals transmitted between the gut microbiota and the brain via neural, endocrine, immune, and humoral pathways.

*The Second Brain Hypothesis* highlights the gut's extensive neural network—known as the ENS—which operates with a degree of autonomy and is sometimes referred to as the “second brain.” While this concept overlaps with the gut–brain axis hypothesis, which focuses on bidirectional communication between the gut and CNS via the vagus nerve, the second brain hypothesis emphasizes that the gut itself can independently sense, process, and respond to stimuli without direct input from the brain. The vagus nerve (cranial nerve X), one of the body's longest and most complex nerves, connects the brainstem to the abdomen and regulates parasympathetic functions such as heart rate, digestion, and respiration (48). This bidirectional signaling via the vagus nerve represents a direct example of the gut–brain axis hypothesis, illustrating how the brain affects gut function and, conversely, how gut-derived signals can influence brain activity.

Recent studies have shown that vagus nerve activity and stimulation (VNS) can modulate mood and cognition, supporting its role as a communication pathway within the gut–brain axis (49, 50). However, the ENS's intrinsic neural circuits are capable of local decision-making—coordinating gut motility, secretion, and immune responses—independently of central control. This semi-autonomous function supports the notion that the gut can act as a “second brain.” Together, these perspectives suggest that both autonomous ENS function and vagus-mediated signaling contribute to the gut's influence on emotional and cognitive health, offering new therapeutic targets for neurological and psychiatric disorders (49, 50).

The gut-brain axis, the second brain, and microbiome-immune modulation are not mutually exclusive theories. Rather, they most likely work together in a coordinated network that promotes cognitive development, with HMOs influencing the enteric and central neurological systems, modifying immune responses, and influencing the gut microbiota in parallel. The vagus nerve is a vital conduit among these routes, converting information from the gut into central brain responses, underscoring its critical function in moderating the effects of HMOs.

## The function of the vagus nerve assessment

Several methods can be employed to assess vagus nerve function. VNS applies electrical impulses to the vagus nerve in animal models to observe changes in gut function, brain activity, and behavior (48). Vagal lesioning involves surgically cutting or blocking specific branches of VN to examine its role in gut-brain communication (51). Microbiome analysis compares gut bacteria in animals with vagal stimulation or lesions to controls, providing insight into how VN activity influences the gut microbiota (52, 53). Brain imaging techniques like functional MRI (fMRI) and PET scans visualize brain activity changes in response to VN modulation *in vivo*. Neurotransmitter analysis measures neurochemical changes, such as glutamate, GABA, and glycine, in the brain and gut (54). Behavioral assessments, including the open field test, elevated plus maze, and forced swim test, can evaluate mood, anxiety, stress responses, and other behaviors affected by VN manipulation. Vagal nerve recording uses electrodes to monitor VN activity in response to gut stimuli. In humans, transcutaneous vagus nerve stimulation (tVNS) offers a non-invasive method to study vagal activity's impact on the gut-brain axis (55). In this review, we discusshow the nervous system, particularly the VN, regulates overall health and highlights its potential as a therapeutic target. However, the function of the vagus nerve is typically assessed using physiological, electrophysiological, or behavioral indicators, depending on the research or clinical context. Further investigation is warranted, as the intricate structural and functional organization of the gut and brain precludes direct measurement of their interactions. Consequently, most existing evidence is based on indirect assessments, posing a major limitation to current understanding.

## Microbial impact on the pig and human gut

The gastrointestinal tract (GIT) hosts a complex and intricate ecosystem comprised of trillions or billions of bacteria from over a thousand taxonomic groups, along with viruses, archaea, fungi, and other eukaryotic species, collectively known as the microbiome (56). This diverse microbiome plays vital roles in GIT functions, though many species remain uncultured and unidentified. While genetics shape the microbiome, early-life exposures, such as delivery mode and breastfeeding, have a significant influence (57).

Human milk contains a diverse microbiome essential for seeding the infant's gut (58, 59). Culture-based and genomic techniques have revealed approximately 820 bacterial species in human breast milk, dominated by *Firmicutes* and *Proteobacteria*, with a core bacteriome comprising *Staphylococcus* spp., *Streptococcus* spp., and others (60). Fungal presence, primarily *Candida, Alternaria*, and *Rhodotorula*, have also been detected, though they are often overlooked (61). Viruses, including bacteriophages, are transmitted from mother to infant via breastfeeding, influencing the neonatal virome and potentially benefiting bacterial populations (62). Environmental factors and diet, such as different milk formulas, further shape its composition, thereby impacting health. Most microbes are symbiotic, benefiting both host and microbe, while some can be pathogenic.

Pigs are valuable models for biomedical research due to their microbiome similarity to humans. Their gastrointestinal tract hosts ~10^14^ bacterial cells, which is 2 to 10 times greater than the estimated number of host cells (63, 64). In a previous study, the collective genes of the bacterial population in the pig gut were estimated to be around 17.2 million, surpassing the pig genome (~25,000 genes) by over 680 times (65). Due to the significant resemblance in omnivorous behavior and gastrointestinal structure between humans and pigs, pigs have emerged as crucial animal models for investigating the gut microbiome. Some studies have compared the gut microbial compositions between humans and pigs using gene catalogs of the human and pig gut microbiome (65, 66). They reported that the proportion of shared bacterial genera between humans and pigs (87.24% for human gut microbiota) exceeded that shared between humans and mice (70.00% for human gut microbiota) (65). Additionally, the alpha diversity of the pig gut metagenome was higher than that of the mouse and human microbiomes, as reported in comparative metagenomic studies (65, 67). Significant variations were observed in the abundance of specific bacterial species across different regions of the gastrointestinal tract. The human and pig microbiomes share about 9.5% of their microbial species, and there is a 96% overlap in the functional pathways of biological processes between humans and pigs, supporting the use of pigs as valuable biomedical models (67). These findings demonstrate that the microbiota composition of pig feces more closely resembles that of humans than that of rodents (68). Importantly, while common bacterial genera are present in both human and pig gut microbiomes, their abundances may differ. For example, pig feces are more likely to contain *Lactobacillus* and *Clostridium* than human feces, where *Bacteroides* and *Eubacterium* predominate at the genus level (65).

To achieve a comprehensive understanding of overall gut health and systemic effects, it is advisable to include samples from multiple gut segments, such as the ileum, cecum, and rectum. This approach provides a broader perspective on the diverse microenvironments and functions within the gut. Selecting specific segments based on their unique characteristics can yield targeted insights: the ileum, involved in nutrient absorption, is suitable for studying small intestinal microbiota (69, 70); the cecum, characterized by high microbial diversity and fermentation activity, is ideal for investigating fermentation products and microbial diversity (71) and the rectum, associated with final waste processing, is pertinent for examining large intestinal microbiota (72, 73). This multi-segment approach allows for a more nuanced and comprehensive analysis of the gut's complex ecosystem, thereby enhancing our understanding of its roles in health and disease.

There is a distinct difference in microbial composition among ileum, cecum, and colon in pig and humans (65). Although the microbiomes in different gut segments of pigs and humans vary, studying the changes in the pig gut microbiome remains highly relevant for human research. By examining how HMOs function in pigs, we can gain insights into similar mechanisms that might occur in humans. Comparing the effects of HMOs observed in pigs with those seen in human studies helps validate the consistency of results. Additionally, findings from pig studies can be applied to laboratory experiments with human samples to assess their feasibility. This comment can then support clinical trials to verify the effects of HMOs in humans. Utilizing results from pig studies can help to provide a more robust scientific basis for human research.

The composition of gut microbial communities varies across different sections of the gastrointestinal tract, such as the ileum, cecum, and feces in both animals and pigs. Each segment provides a distinct microenvironment shaped by nutrient availability, pH, oxygen concentration, and transit time. The ileum is characterized by relatively low microbial density and is dominated by facultative anaerobes that participate in nutrient absorption and bile acid metabolism. In contrast, the cecum exhibits high microbial diversity and intense fermentative activity that produces SCFAs, while the rectum functions as a terminal fermentation site and largely mirrors the fecal microbiota composition (70, 74) HMOs reach the distal intestine largely intact, where they are selectively fermented by bifidobacteria and lactobacilli, leading to compositional and metabolic changes in these regional microbial communities (75). Segment-specific fermentation models further demonstrate that HMOs such as 2′-fucosyllactose enhance SCFA production and modulate microbial metabolic pathways relevant to host immunity and intestinal barrier function (76). Several studies have already manipulated pig diets with defined HMOs to investigate how these glycans alter gut microbial composition and metabolism. For example, supplementing piglet formula with 2′-fucosyllactose or blends of 3′- and 6′-sialyllactose significantly increased colonic SCFA levels, enriched bifidobacteria and lactobacilli, and promoted intestinal maturation (76, 77). Similar results were observed in preterm pig models, where multi-HMO supplementation altered colonic microbiota composition and the metabolic fate/excretion profile of HMOs (77). The gut microbiota in pigs comprises several dominant microbial groups along with a smaller number of less common microorganisms. Researchers can modify the composition and amounts of specialized HMOs in pig diets, thereby altering these microbial habitats. Through manipulation of the pig's diet, scientists may track changes in the gut microbiome and evaluate the interaction between these microbial communities and HMOs. This enables them to determine how HMOs influence and interact with microbial communities. Clinical research can then apply the findings from pig studies to explore if HMOs affect the human gut microbiota comparably. If these results hold true for human participants, researchers will be able to evaluate the potential of HMOs to improve gut health. Previously we demonstrated that dietary sialyllactose promotes intestinal maturation in neonatal piglets by upregulating GDNF, synthesis of polySia and CREB-interactive pathway (78). This research avenue may lead to the development of HMO-based dietary therapies aimed at enhancing human gut health and associated physiological functions. Furthermore, this approach provides new insights into human health in addition to assisting understanding the complex relationships between the microbiota and the host. For example, insights into how HMOs influence the microbiome in pigs can lead to the development of novel preventive and therapeutic strategies to improve human gut health. Therefore, research on the pig gut microbiome holds great potential and practical value in advancing scientific progress in human health.

## Physiological effects of HMOs related to gut

HMOs support diverse physiological functions. HMOs are intricate carbohydrates that serve as a prebiotic fuel for the developing gut microbiota in infants (79). HMOs have valuable effects in maintaining a stable gut ecosystem in infants, which include shaping intestinal microbiota, imparting antimicrobial effects, developing the intestinal barrier, and modulating immune responses (10, 57). HMOs serve as selective substrates that promote the growth of beneficial bacteria such as *Bifidobacterium* and *Lactobacillus* species, which contribute to immune maturation, gut barrier integrity, and pathogen resistance (80). However, the composition of milk microbiota and the abundance of specific HMOs can vary among individuals, and this interaction may be influenced by factors such as maternal genetics, diet, mode of delivery, and environmental exposure (81). HMOs can act as selective substrates for beneficial bacteria, such as *Bifidobacteria*, promoting their growth and inhibiting the proliferation of harmful pathogens (82). This intricate interplay contributes not only to the establishment of a diverse and balanced gut microbiome but also to the development of a robust immune system. HMOs exert multiple physiological effects on the gastrointestinal tract through their selective interaction with the gut microbiota and intestinal epithelium. Acting as prebiotic substrates, HMOs promote the growth of beneficial bacteria such as *Bifidobacterium longum* subsp. *infantis* and *Bacteroides fragilis*, while suppressing opportunistic pathogens including *Clostridium difficile* and *Escherichia coli* (80). Beyond their metabolic effects, SCFAs and HMO-derived metabolites contribute to intestinal immune maturation by modulating dendritic cell activation, enhancing mucosal IgA production, and promoting the differentiation of regulatory T cells (83). HMOs also protect against infection by blocking pathogen adhesion to epithelial glycoconjugate receptors and strengthening tight-junction integrity (84). Collectively, these mechanisms illustrate how HMOs orchestrate the gut environment to favor beneficial microbial colonization, maintain epithelial homeostasis, and support host immune balance during early life.

## HMOs and behavior

Studies have shown that HMOs can significantly impact animal models' behavior significantly, especially in areas such as social interactions, anxiety, and cognitive functions (1, 85). Studies have shown that early intake of 2′-FL can improve learning and memory by supporting brain development. In neonatal pig models, 2′-FL supplementation enhanced hippocampal gene expression related to synaptic plasticity and increased sialic acid levels, which are important for neural connectivity and cognition. These findings suggest that 2′-FL contributes to cognitive development by promoting neuronal growth and function rather than general “brain health” (86, 87). These effects may be partly mediated through the gut microbiota, as beneficial strains such as *Bifidobacterium longum subsp. infantis*—which efficiently metabolizes 2′-FL—are commonly enriched in breastfed infants. Breastfed infants often show better cognitive outcomes than formula-fed infants, and this advantage is partly linked to the unique HMOs in human milk that shape the gut–brain axis (88). While HMOs may reduce stress-related behaviors in pigs through modulation of gut microbiota and enrichment of beneficial bacteria, direct evidence of reduced anxiety-type behaviors in pig HMO supplementation trials is still limited. In pigs, anxiety-associated behaviors such as trembling, vacuum chewing, yawning or itch-scratch cycles have been described in social stress paradigms (89). This shift in the microbiota may lead to an increased production of SCFAs, such as butyrate, which are known to influence stress and mood regulation in other animal models. However, anxiety-like behaviors have not been directly evaluated in pigs, and this potential link remains hypothetical (90, 91). In animal models, supplementation with HMOs such as 2′-FL or sialyllactose has been linked to improved social and exploratory behaviors, likely through modulation of gut microbiota and increased *Bifidobacterium* and *Lactobacillus* abundance that produce neuroactive metabolites like GABA and serotonin (the Combination of 2′-Fucosyllactose with Short-Chain Galacto-Oligosaccharides and Long-Chain Fructo-Oligosaccharides that Enhance Influenza Vaccine Responses Is Associated with Mucosal Immune Regulation in Mice; Human and Bovine Milk Oligosaccharides Elicit Improved Recognition Memory Concurrent With Alterations in Regional Brain Volumes and Hippocampal mRNA Expression). These behaviors were evaluated using open-field and novel-object tests (92, 93).

Multiple studies have reported that specific HMOs enhance learning and memory performance. In pig models, supplementation with 2′-FL and sialyllactoses (3′-SL, 6′-SL) improved spatial learning and memory retention in T-maze and novel object recognition tasks, reflecting better working memory and exploratory behavior (33). Similar findings were observed in rodent studies where 2′-FL and 3′-FL supplementation enhanced recognition and spatial learning performance (94). The proposed mechanisms involve reduced neuroinflammation and increased neurotrophic signaling, possibly mediated by microbiota-derived metabolites such as short-chain fatty acids that can cross or influence the blood–brain barrier (BBB) to support neuronal function (95, 96).

Although multiple observational and interventional studies in breastfed infants and children have shown beneficial associations between early gut-microbiota modulation and cognitive outcomes, an increasing number of human trials have now investigated direct HMO supplementation. Willemsen et al. reported that higher intake of fucosylated HMOs during early infancy was correlated with better executive function by the age three (97). Other randomized controlled trials using specific HMOs such as 2′-FL, LNnT, 3′-SL, and 6′-SL, alone or in combination with probiotics (Bifidobacterium infantis, B. breve), have shown benefits for gut microbiota composition, immune maturation, and overall health outcomes in infants (25, 86, 98, 99).

Behavioral and neurodevelopmental assessments in these studies commonly included measures of executive function, memory, language, and motor skills, revealing associations between higher HMO exposure and improved cognitive performance. Further large-scale studies are needed to clarify how different types and combinations of HMOs influence specific human cognitive functions through gut–brain axis mechanisms involving microbial metabolites, short-chain fatty acids, and vagus-nerve signaling.

## Translating HMOs into infant formula

HMOs have successfully been incorporated into infant formula in several countries. For instance, 2′-FL and LNnT have been approved for use in infant formula and are produced via microbial fermentation using genetically engineered E. coli or yeast strains that express human glycosyltransferases (100). These enzymes catalyze the stepwise transfer of monosaccharides to lactose, producing specific HMOs such as 2′-FL, 3′-FL, 6′-SL, and LNnT. The fermentation products then undergo purification through filtration and chromatography to ensure safety and purity before being added into formula. The number and types of HMOs approved for use vary across regions. Beyond 2′-FL and LNnT, other HMOs, such as 3′-FL, 6′-SL, and LNFP, are being studied for commercial use (101). In addition to microbial fermentation, enzymatic or chemo-enzymatic synthesis is being explored as an alternative production route, offering higher structural precision though at higher cost. Commercial infant formulas currently include 0.2–2.4 g/L of HMOs, aiming to mimic the lower end of natural human milk levels. However, multi-HMO formulations require further research to determine their combination effects. HMOs exert dose-dependent effects on the gut microbiome, immune modulation, and cognitive development, with evidence suggesting potential threshold or saturation effects (102, 103). Their inclusion in infant formula seeks to replicate the benefits of human milk, but the optimal composition and dosage remain under investigation. Future research should focus on refining dose-response relationships to optimize HMO's nutritional and therapeutic applications.

## Challenges and gaps of HMOs research

Despite promising findings, several challenges and gaps remain in HMO research. Many studies feature small sample sizes and short durations, limiting the ability to assess the long-term effects of HMOs on gut microbiota composition and cognitive function. Advanced omics techniques, including metagenomics, metabolomics, and single-cell omics, have already been widely applied to investigate how HMOs influence gut microbial composition and metabolic activity, revealing key taxa and pathways involved in short-chain fatty acid production and immune modulation (104, 105). However, integrative multi-omics approaches combining these datasets with host transcriptomics or neurochemical profiling could further elucidate the complex gut–brain mechanisms underlying HMO effects. A multi-omics approach is essential in gut microbiome research because it enables a comprehensive investigation of the intricate relationships between the microbiome and the host across multiple biological layers. Additionally, Fecal microbiota transplantation (FMT) has already been applied in studies investigating how HMOs influence gut microbial composition and function (106). However, most existing FMT experiments have been conducted in rodents, and further development of FMT models in more translatable species such as pigs would be valuable for pre-clinical evaluation before human trials.

These models simulate realistic microbial transplantation conditions, offering critical insights into how HMOs alter or modify the gut microbiome. By integrating these methodologies, scientists can more accurately evaluate the potential of HMOs to regulate gut microbiota and improve host health. This approach will generate stronger experimental evidence to support future human clinical trials.

Furthermore, a deeper understanding of how different types and dosages of HMOs affect pig behavior will enable more precise dietary recommendations for pigs and other species. Previous pig studies have mainly examined 2′-FL, 3′-SL, and 6′-SL, either individually or in combination, showing effects on cognition, stress response, and gut microbial composition (33, 107). However, the number of pig studies that include detailed behavioral assessments across multiple HMO types and dosages remains limited, which constrains our ability to determine optimal formulations or standardized protocols.

The complexity of HMO structures, which include various subtypes such as fucosylated and sialylated HMOs, requires systematic investigations that compare these distinct HMOs under carefully controlled conditions. Furthermore, precisely determining the optimal dosage to effectiveness ratio is essential for understanding how these compounds affect gut microbiota and behavior. By refining these variables, future research can establish more precise standards, paving the way for evidence-based nutritional therapies suitable for both humans and livestock.

Despite increasing interest in the health benefits of HMOs, one of the challenges remains their high production cost, primarily driven by the structural complexity of HMOs that renders chemical synthesis inefficient and costly. Additionally, scalability poses significant hurdles, as methods that perform well at laboratory or pilot scales often struggle to adapt to industrial-scale manufacturing. Intellectual property rights and patent restrictions related to HMO biosynthesis strains and enzymes further constrain commercial freedom, creating barriers for new market entrants. Moreover, with rising consumer awareness, there is growing demand for sustainable and cost-effective production approaches, underscoring the need for greener and more economical manufacturing technologies.

## Conclusion

This review concludes by underscoring the potential of HMOs as significant modulators of both gut health and cognitive function. Further mechanistic studies are needed to elucidate the interactions among HMOs, the gut brain axis, and microbial metabolites to develop targeted nutritional interventions that optimize physiological and neurocognitive outcomes. While current research predominantly addresses HMOs in human infant nutrition, emerging evidence supports the investigation of HMOs in animal models, particularly piglets, due to their physiological and microbiome similarities to humans. The key question is whether HMOs confer comparable functional benefits across species. In current research, pig models are primarily used for biomedical purposes to evaluate the translational relevance of HMOs to human health, rather than for agricultural application. These studies provide valuable insights into gut, immune, and neurodevelopmental mechanisms that may inform human nutrition research. It is not intended that HMOs be used as dietary supplements for livestock.

In conclusion, although current findings on the role of HMOs in human nutrition are encouraging, the precise mechanisms by which HMOs influence cognitive performance and gut function remain insufficiently understood. Most existing studies have focused on individual HMOs, such as 2′-FL or LNnT, even though human breast milk contains over 200 structurally diverse oligosaccharides. LNnT differs structurally from 2′-FL by its N-acetyllactosamine backbone rather than a fucosylated lactose core, which may lead to distinct microbial utilization patterns and functional effects. Recent evidence suggests that LNnT can promote the growth of Bifidobacterium species and support gut barrier integrity in infants (22, 26). The optimal composition, synergistic combinations, and effective dosages of HMOs for formula-fed infants—as well as their potential benefits across the human lifespan—have yet to be fully elucidated.
